# Eclampsia, or, Puerperal Convulsions

**Published:** 1881-10

**Authors:** D. A. Walden

**Affiliations:** Beatrice, Neb.


### ARTICLE IV.

### ECLAMPSIA, OR PUERPERAL CONVULSIONS. By D. A. WALDEN, M.D., Beatrice, Neb.

In May, 1879, I was called to see Mrs. H., a primipara, age 23 years, in her seventh month of gestation. Found her in puerperal convulsions, which would last from one to five minutes, and each paroxysm was followed almost immediately by another.

I remained with the patient all night, and administered chloroform by inhalation, but could not control the convulsions. In the morning I began to administer morphia sulph. in one gr. doses, but with no better results. I followed this with hydrate chloral, 60 grains every two hours, and bromide potass., 80 grains every three hours, and continued this treatment for forty-eight hours, without controlling the convulsions in the least.

Counsel was called, and a delivery was concluded to be the only means of saving life. Fœtus was dead—woman made perfect recovery.

In March of the year 1881 the same woman was taken, in her eighth month of pregnancy, with puerperal convulsions the second time. I was called to see her, and found her almost as before; gave her the same remedial treatment as before, but without any good results.

The idea occurred to me: would not quinia have good effect in such cases? I concluded to try it, at least, as I had tried all remedies that I could find in books on that disease. I wrote a prescription for quinia sulph., grs. 80, to be divided into three powders, one to be given every two hours.

After an hour from the time the first powder was taken I could see a change; the convulsions were becoming lighter. Continued the quinia until six 10 gr. powders were taken, at which time the convulsions were entirely controlled, and did not again return.

The patient went to her full term, and then I delivered her of a healthy female child. The mother and child are doing well up to this date.

Case second. Mrs. B., a multipara, age 24 years, in her eighth month of pregnancy, was taken with puerperal convulsions on July 8, 1881. I was called to see her; found her in convulsions most of the time; remained with her all night, and gave chloroform by inhalation without any effect. Then remembering my former case, I ordered quinia sulph., grs. 60, divided into six powders, one to be taken every two hours. A change was noticed in about an hour after the first dose was taken, and the convulsions had ceased entirely by the time the six doses were taken, but came on again the next day. I then ordered the quinine again, and continued it for three days.

The convulsions did not again appear, the patient went to her full term, and I then delivered her of a healthy male child.

Case third did not come directly under my notice, yet I will cite it, to further prove that there is really some virtue in quinia for this disease.

After trying quinia in my first case, I related my experience to Dr. S. G. Huff, of this place, and soon after, he, having a case of the same kind, thought, after trying the usual remedies of bromide potass., chloral hydrate, chloroform, etc., that he would give the quinia treatment a trial, as the more that bromide potass., chloral hydrate, etc., were given, the worse the patient became. The convulsions were so severe that the assistance of two or three persons was required to keep the patient in bed.

He put her on 10 gr. doses of quinia, one to be given every two hours; it controlled the convulsions completely, and she has not had a return of them since. Her entire term of gestation is now nearly up.

I will not enter into the pathological condition of puerperal convulsions, for that has been fully discussed by all of our eminent authors, yet I would say to the profession that it may be of some interest and benefit to give quinine a fair trial in this disease.

